# Selection and validation of reference genes for the normalization of quantitative real-time PCR in different muscle tissues of rabbits

**DOI:** 10.1186/s40850-022-00159-0

**Published:** 2022-12-15

**Authors:** Mengke Ni, Zhichao Li, Jing Li, Hui He, Yaling Wang, Yixuan Jiang, Xianwei Wang, Zhuanjian Li, Ming Li, Huifen Xu

**Affiliations:** 1 College of Animal Science and Technology, Henan Agricultural University, Zhengzhou, 450046 P. R. China; 2 Animal Health Supervision Institute of Biyang, Henan, 463700 P. R. China; 3 Henan Provincial Animal Husbandry General Station, Zhengzhou, 450008 P. R. China

**Keywords:** Reference genes, Muscle, Rabbits, Quantitative real-time PCR

## Abstract

**Background:**

In molecular biology studies, the selection of optimal reference genes is of vital importance for accurately quantifying gene expression. The purpose of the present study was to screen the most stable reference genes in different muscle tissues of New Zealand white rabbits and Yufeng yellow rabbits.

**Methods and results:**

Results indicated that the most stable reference genes in the muscle tissues of New Zealand white rabbits were *HPRT1*, *ACTB* and *PPIC*, while *HPRT1*, *PPIC*, and *RPL13A* were the most stable reference genes in muscle tissues of Yufeng yellow rabbits. However, in the *longissimus dorsi* muscle and the abdominal wall muscle of both varieties, the most stable reference genes were *HPRT1*, *RPL13A*, and *SDHA*. In the *quadriceps femoris* muscle, the most stable reference genes were *ACTB*, *HPRT1*, and *SDHA*. Furthermore, the relative abundance of *MYOG*, *MYH3* and *MSTN* was used to confirm the suitability and reliability of the selected most stable reference genes and the most unstable reference gene. Results revealed the same expression patterns of these myogenic genes when normalized according to the most stable genes, while normalization against the unstable reference gene altered the observed expression patterns.

**Conclusions:**

Taken together, our results demonstrated that the most stable reference genes varied among different muscle tissues and different breeds of rabbits. However, *HPRT1*, *PPIC* and *SDHA* presented high stability among all examined reference genes; thus, the combined analysis of *HPRT1*/ *PPIC*/ *SDHA* gene provides the best reference for RT-qPCR in muscle tissues of New Zealand white rabbits and Yufeng yellow rabbits, while *HPRT1* is a better choice than other reference genes when using a single reference gene to assess target gene expression. Our results provide basic data for better measuring target gene expression profiles in muscle tissues of rabbits.

**Supplementary Information:**

The online version contains supplementary material available at 10.1186/s40850-022-00159-0.

## Background

With the development of molecular biology technologies, the quantification of gene expression has been performed with omics technologies at different levels. However, reverse transcription quantitative real-time PCR (RT-qPCR) remains the most commonly used method to measure the transcriptional abundance of target genes due to its high efficiency, sensitivity and specificity. The reliability of RT-qPCR depends on the normalization of mRNA abundance by using selected, stably expressed reference genes. Thus, the selection and use of stable reference genes in RT-qPCR analyses are key for determining the accurate expression patterns of genes.

Stable reference genes have been reported in many studies in humans and animals. *GNB2L1*, *HPRT1*, *RPL32*, *ACTB* and *B2M* were demonstrated to be the most stable reference genes in a screen of expression patterns in human neutrophils [1]. PPIA and TBP were selected as ideal reference genes for the standardization of gene expression in mouse liver [2]. Studies of cattle muscle development showed that *SF3A1*, *EEF1A2* and *HMBS* were suitable reference genes [3]. Goossens et al. showed that *YWHAZ*, *GAPDH* and *SDHA* were suitable reference genes for bovine preimplantation embryos [4]. These results showed that suitable reference genes are differ among different species and treatment conditions.

To date, the most commonly used reference genes in rabbits include actin beta (*ACTB*), peptidylprolyl isomerase C (*PPIC*), glyceraldehyde-3-phosphate dehydrogenase (*GAPDH*), beta-2-microglobulin (*B2M*), succinate dehydrogenase complex flavoprotein subunit A (*SDHA*), hypoxanthine phosphoribosyltransferase 1 (*HPRT1*), ribosomal protein L13a (*RPL13A*) and 18S ribosomal RNA (*RN18S*). These reference genes are also known as “housekeeping genes” because of their important role in maintaining the basic physiological functions of cells. Among these genes, *ACTB*, *GAPDH*, *HPRT1*, *RN18S* and other reference genes have been widely used for northern blot hybridization, conventional PCR analysis and semiquantitative analyses [5, 6]. The expression of these reference genes has long been considered to be stable in different tissues and experimental conditions. However, increasing evidence has shown that the expression of some commonly accepted reference genes, including *GAPDH* and *ACTB*, varies in different tissues, cells, and developmental stages [7–9]. Therefore, it is advisable to select appropriate reference genes under different conditions, and the selection of suitable reference genes in scientific research is critical for obtaining reliable and accurate results.

However, there is few reports about stable reference gene analysis in rabbit muscle tissues [10, 11]. In the present study, eight genes including *PPIC*, *HPRT1*, *ACTB*, *RPL13A*, *GAPDH*, *SDHA*, *RN18S* and *B2M* were selected as candidate reference genes, and three programs (geNorm, NormFinder, and BestKeeper) were used to evaluate the stability of these candidates as reference genes [12–15]. This study aimed to identify the most stable reference genes in muscle tissues of rabbits, and the mRNA expression of myogenin (*MYOG*), myosin heavy chain 3 (*MYH3*) and myostatin (*MSTN*) was quantified in rabbit muscle tissues to validate the stability of the selected reference genes. The assessment of reference gene stability will be beneficial for more accurately measuring target gene expression profiles in rabbit muscle tissues.

## Methods

### Animals and tissue samples

In this experiment, the rabbits were purchased from rabbit breeding grounds of Yufei, Zhengzhou, China. Five male New Zealand white rabbits (70-day-old, 2.37 ± 0.046 kg) and five male Yufeng yellow rabbits (70-day-old, 2.63 ± 0.158 kg) were selected. Rabbits used in the present study were randomly selected and they did not come from the same pair of parents and did not relate with each other. A randomized outbreeding protocol is used to maintain genetic diversity. Rabbits were raised in separate cages (35 cm × 45 cm × 45 cm) and kept the temperature at 20–25 °C under a 12 h: 12 h (light: dark, lights on at 08:00 h) photoperiod. All rabbits were in good health and provided ad libitum access to commercial pellet diets (probiotics and antibiotics free) and water. Rabbits were sacrificed using an overdose of isoflurane (Abbot, Chicago, IL, USA). After slaughter, the *longissimus dorsi* muscle, abdominal wall muscle and *quadriceps femoris* muscle tissues were collected and frozen in liquid nitrogen until analysis.

### RNA isolation and cDNA synthesis

Total RNA isolation was performed by using TRIzol (TransGen, China) according to the manufacturer’s instructions. The qualitative and quantitative assessment of the isolated RNA was performed using a spectrophotometer (NanoDrop One, ThermoFisher Scintific, USA). RNA integrity was assessed by the electrophoretic analysis of 28S and 18S rRNA subunits (Supplementary Fig. S4). Qualified RNA (A260/280 = 2.0; A260/230 = 2.1) was reverse transcribed into cDNA using the PrimeScript™ RT reagent Kit with gDNA Eraser (Perfect Real Time) (TaKaRa, RR047A, Japan). 1000 ng of RNA and random 6 mers or oligo (dT) Primer were used in each 20 μL volume RT reaction. The resulting 20 μL cDNA product is diluted to a final volume of 80 μL before qPCR.

### Quantitative real time RT-PCR (RT-qPCR)

The CFX96™ Real-Time System was used for RT-qPCR with ChamQ™ Universal SYBR® qPCR Master Mix (Vazyme Biotech, China). The 10 μL reaction consisted of 5 μL ChamQ™ Universal SYBR® qPCR Master Mix, 0.2 μL forward primer, 0.2 μL reverse primer, 2 μL cDNA, and 2.6 μL ddH_2_O. In addition, the concentration of the primers was 10 pmol/μL. The reaction was performed as follows: 95 °C for 30 sec to activate the polymerase, followed by 40 cycles of denaturation at 95 °C for 10 sec, annealing at 60 °C for 30 sec and extension at 72 °C for 30 sec. Melting curve analysis was performed from 65 °C to 95 °C with 0.5 °C increase every 5 sec to determine amplification specificity. RT-qPCR was performed consecutively without interruption. Tissue samples used for the validation experiment were the same as the gene stability experiment, and all treatments were performed with five biological replicates and three technical replicates. Gene specific primers of *PPIC*, *HPRT1*, *ACTB*, *RPL13A*, *GAPDH*, *SDHA*, *RN18S*, *B2M*, *MYOG*, *MYH3*, and *MSTN* genes were designed by using Primer 6.0 software according to rabbit gene sequences published in GenBank (NCBI, USA). Primers (Supplementary Table S1) were synthesized by Sangon Biotech Co., Ltd. (Shanghai, China) and were optimized before the initial screening and quantitation experiments.

### Analyses of reference gene primers

To calculate PCR efficiency, standard curves were generated from assays conducted with five-fold serial dilutions (5^0^, 5^− 1^, 5^− 2^, 5^− 3^, and 5^− 4^) of six pooled cDNA preparations from different muscle tissues (ng/μL). To ensure the compatibility of the PCR assays, three independent serial dilutions were evaluated, which enabled us to determine the R^2^ values and PCR efficiency (*E*) of the individual assays and calculate the correlation between them.

### Data analysis

Calibration curves were drawn with the logarithm of the sample concentration as the in-dependent variable and Cq as the dependent variable. IBM SPSS Statistics 26.0 software was used to calculate the calibration curves of the primers and to test the degree of fitting for the regression equation for R^2^ calculation. RT-qPCR efficiencies (E) were calculated with the eq. E = 10^(− 1/slope)^-1 to determine whether the candidate reference gene primers could be used for RT-qPCR analysis.

LinRegPCR (version 2021.2) [16] was used to normalize Cq values by amplification efficiency (E). Analyses of the gene expression stability across different rabbits and muscle tissues were performed using the geNorm (version 3.5), NormFinder (version 20) and BestKeeper (version 1) programs. The most stable and unstable reference genes were selected to validate the expression of *MYOG*, *MYH3* and *MSTN* by the 2^-ΔΔCq^ method. Gene expression stability was assessed via one-way ANOVA (with Dunnett’s post hoc where indicated for multiple comparisons). Single-treatment comparisons were conducted with Student’s t-test. Statistical significance was defined as *P* < 0.05.

## Results

### Amplification specificity and primer efficiency

Every gene displayed a single peak profile in the melting curve analysis, and agarose gel electrophoresis results revealed a unique band of the expected size (Supplementary Fig. S1 and S2). The amplification efficiency of eight reference genes ranged from 96.2 to 113.7%, and the coefficient of determination (R^2^) varied from 0.970 to 1.000 (Supplementary Table S2). In conclusion, eight candidate reference genes and three target genes met the requirement of Minimum Information for Publication of Quantitative Real-Time PCR Experiments (MIQE) requirements for use in further experiments.

### Gene expression stability analysis of eight candidate reference genes

The stability of the eight candidate reference genes was determined by using the mathematical algorithms geNorm, NormFinder and BestKeeper. In this experiment, a low average expression stability value in geNorm and NormFinder indicated a more stable expression of the reference genes.

**Fig. 1:**
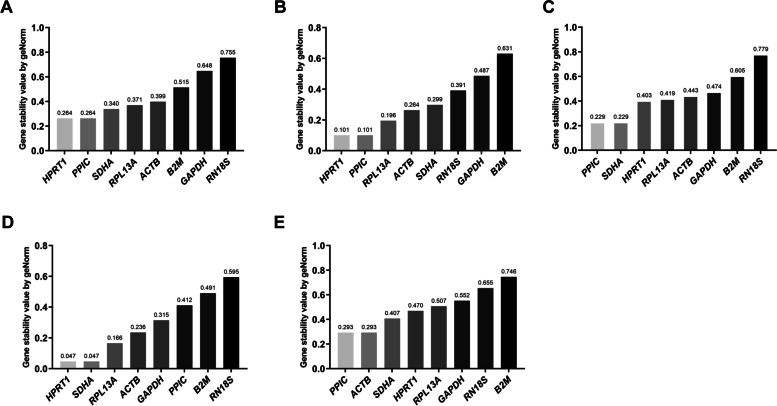
geNorm program analysis results. Expression stability of reference genes in three muscle tissues of New Zealand white rabbits (**A**), three muscle tissues of Yufeng yellow rabbits (**B**), the *longissimus dorsi* muscle tissue of New Zealand white rabbits and Yufeng yellow rabbits (**C**), the abdominal wall muscle tissue of New Zealand white rabbits and Yufeng yellow rabbits (**D**), and the *quadriceps femoris* muscle tissue of New Zealand white rabbits and Yufeng yellow rabbits (**E**)

As shown in Fig. 1, results of the geNorm program showed that the two most stable genes among the three different muscle tissues of New Zealand white rabbits and Yufeng yellow rabbits were *PPIC* and *HPRT1* (Fig. 1A and B). However, stable reference genes were different in different tissues of New Zealand White and Yufeng Yellow rabbits. In the individual tissues of both New Zealand white rabbits and Yufeng yellow rabbits, the most stable reference genes in *longissimus dorsi* muscle tissue were *PPIC* and *SDHA* (Fig. 1C), the most stable reference genes in the abdominal wall muscle tissue were *HPRT1* and *SDHA* (Fig. 1D), and *PPIC* and *ACTB* were the two most stable reference genes in the *quadriceps femoris* muscle tissue (Fig. 1E).

**Fig. 2:**
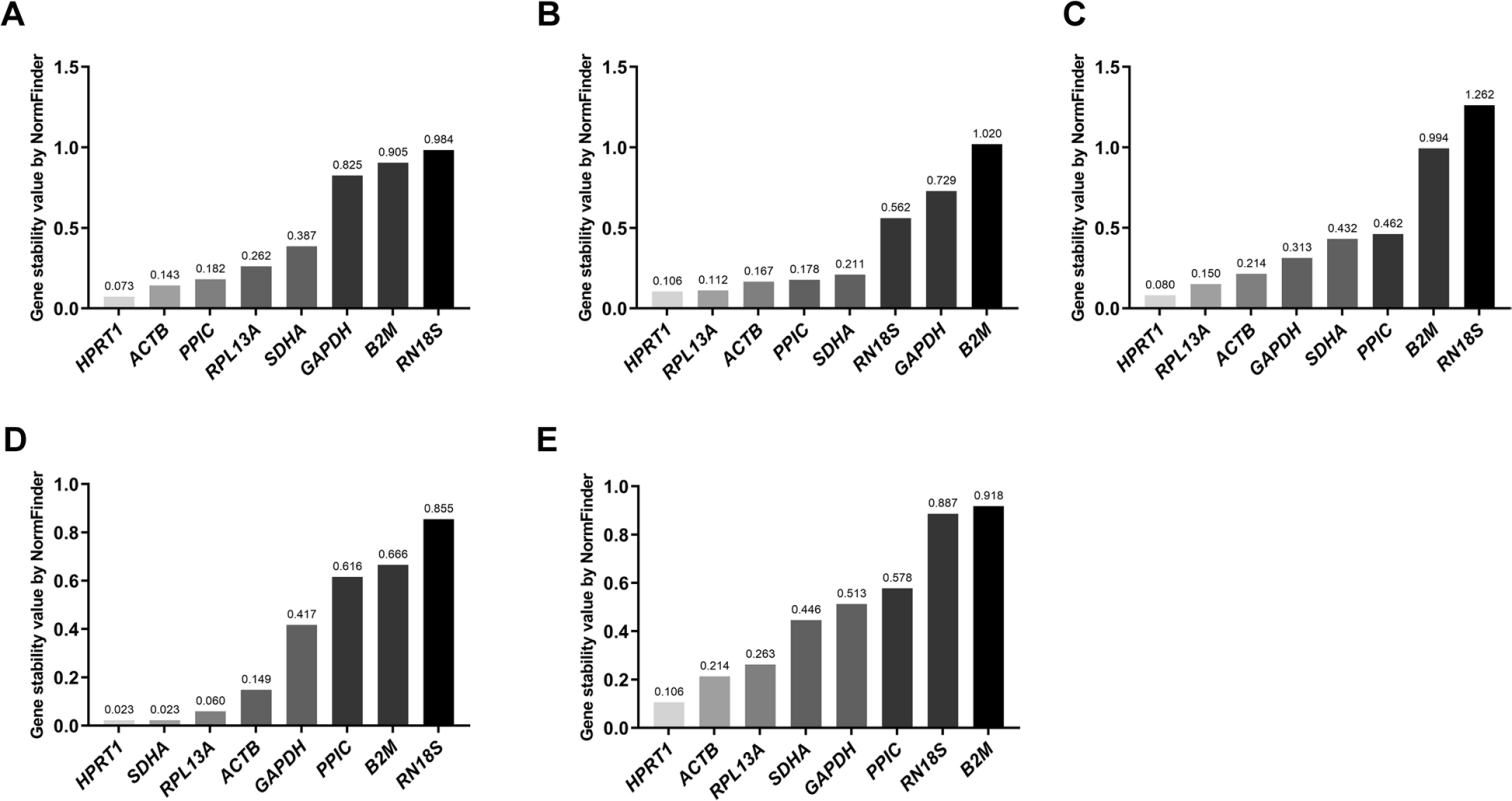
Results of NormFinder analysis. Expression stability of reference genes in three muscle tissues of New Zealand white rabbits (**A**), three muscle tissues of Yufeng yellow rabbits (**B**), the *longissimus dorsi* muscle tissue of New Zealand white rabbits and Yufeng yellow rabbits (**C**), the abdominal wall muscle tissue of New Zealand white rabbits and Yufeng yellow rabbits (**D**), and the *quadriceps femoris* muscle tissue of New Zealand white rabbits and Yufeng yellow rabbits (**E**)

As illustrated in Fig. 2, the results of the NormFinder analysis were generally consistent with those of the geNorm analysis. The only exceptions were that the first-ranked gene in the *longissimus dorsi* muscle and *quadriceps femoris* muscle tissues of both breeds was *HPRT1* (Fig. 2C).

The correlation coefficient (R) and standard deviation (SD) values among the reference genes were acquired via BestKeeper program analysis (Tables 1 and 2). *RN18S* was the most stable reference gene in the muscles of both New Zealand white rabbits and Yufeng yellow rabbits except for the abdominal wall muscle tissue, while the *B2M* gene was the least stable gene. In the abdominal wall muscle, the most stable reference gene was *SDHA*, while *PPIC* was the most variable gene.

**Table 1:** Ranking values of reference genes stability calculated by BestKeeper based on CT value in New Zealand white rabbits and Yufeng yellow rabbits

Rank	New Zealand white rabbits			Yufeng yellow rabbits		
	Gene	Std dev [± CT]	Coeff. of corr.	Gene	Std dev [± CT]	Coeff. of corr.
1	*RN18S*	0.24	0.590	*RN18S*	0.12	0.885
2	*GAPDH*	0.75	0.739	*SDHA*	0.14	0.961
3	*ACTB*	0.79	0.964	*GAPDH*	0.60	0.721
4	*RPL13A*	0.86	0.976	*HPRT1*	0.64	0.963
5	*HPRT1*	0.87	0.968	*PPIC*	0.68	0.955
6	*PPIC*	0.96	0.980	*RPL13A*	0.69	0.964
7	*SDHA*	1.01	0.739	*ACTB*	0.69	0.982
8	*B2M*	1.36	0.920	*B2M*	1.34	0.931

**Table 2:** Ranking values of reference genes stability calculated by BestKeeper based on CT in three different muscle tissues of these two species of rabbits

Rank	***Longissimus dorsi*** muscle			Abdominal wall muscle			***Quadriceps femoris*** muscle		
	Gene	Std dev [± CT]	Coeff. of corr.	Gene	Std dev [± CT]	Coeff. of corr.	Gene	Std dev [± CT]	Coeff. of corr.
1	*RN18S*	0.68	0.625	*SDHA*	0.20	0.786	*RN18S*	0.38	0.421
2	*HPRT1*	0.89	0.979	*HPRT1*	0.21	0.780	*ACTB*	0.61	0.889
3	*GAPDH*	0.90	0.992	*GAPDH*	0.26	0.257	*SDHA*	0.68	0.816
4	*RPL13A*	0.92	0.973	*RPL13A*	0.27	0.834	*HPRT1*	0.69	0.992
5	*SDHA*	1.13	0.895	*ACTB*	0.36	0.915	*PPIC*	0.71	0.753
6	*ACTB*	1.16	0.963	*RN18S*	0.41	0.001	*RPL13A*	0.76	0.985
7	*PPIC*	1.17	0.868	*B2M*	0.53	0.871	*GAPDH*	0.77	0.892
8	*B2M*	1.69	0.907	*PPIC*	0.73	0.813	*B2M*	1.23	0.882

### Combined analysis of the rank order of reference genes according to geNorm, NormFinder, and BestKeeper

**Fig. 3:**
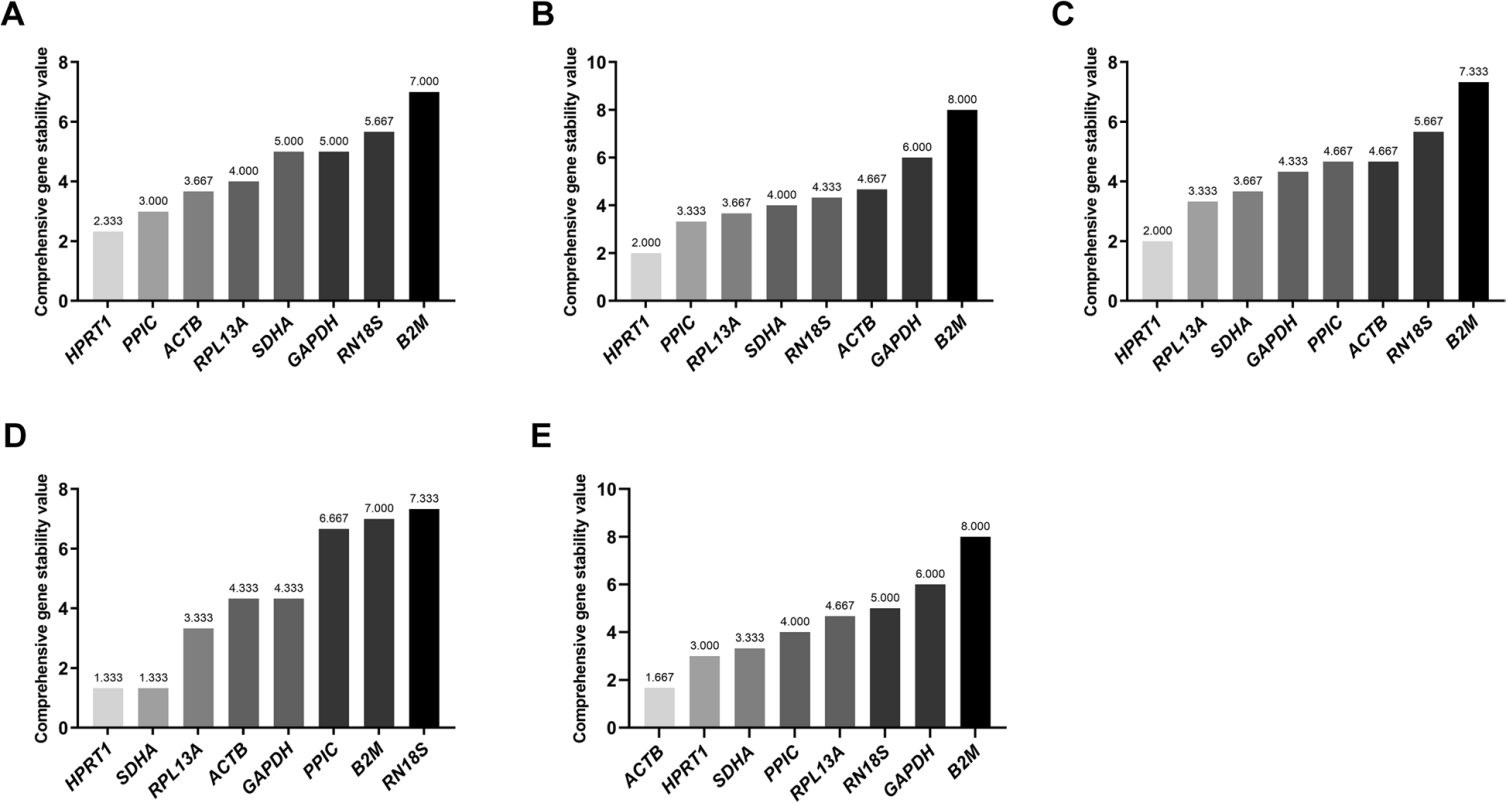
Comprehensive gene stability order. Expression stability of reference genes in three muscle tissues of New Zealand white rabbits (**A**), three muscle tissues of Yufeng yellow rabbits (**B**), the *longissimus dorsi* muscle tissue of New Zealand white rabbits and Yufeng yellow rabbits (**C**), the abdominal wall muscle tissue of New Zealand white rabbits and Yufeng yellow rabbits (**D**), and the *quadriceps femoris* muscle tissue of New Zealand white rabbits and Yufeng yellow rabbits (**E**)

To prevent possible bias, results of the geNorm, NormFinder and BestKeeper program analyses were comprehensively ranked, with the most stable reference genes being ranked 1st. Then, the arithmetic average of each reference gene was calculated, and the stability of the reference genes was comprehensively analyzed. As illustrated in Fig. 3A, *HPRT1* was the most stable reference gene in the three muscle tissues of New Zealand white rabbits, followed by *PPIC* and *ACTB*, while the most stable reference genes in Yufeng yellow rabbits were *HPRT1, PPIC*, and *RPL13A* (Fig. 3B). *HPRT1* was the most stable reference gene in both the *longissimus dorsi* muscle and the abdominal wall muscle tissue of New Zealand white rabbits and Yufeng yellow rabbits (Fig. 3C, D). *ACTB* was the most stable reference genes for the *quadriceps femoris* muscle, followed by *HPRT1* and *SDHA* (Fig. 3E). The algorithms for each differ and, as such, their combined application should improve confidence in the selection of ideal candidate reference genes when there is congruence.

### Evaluation and validation of selected reference genes

**Fig. 4:**
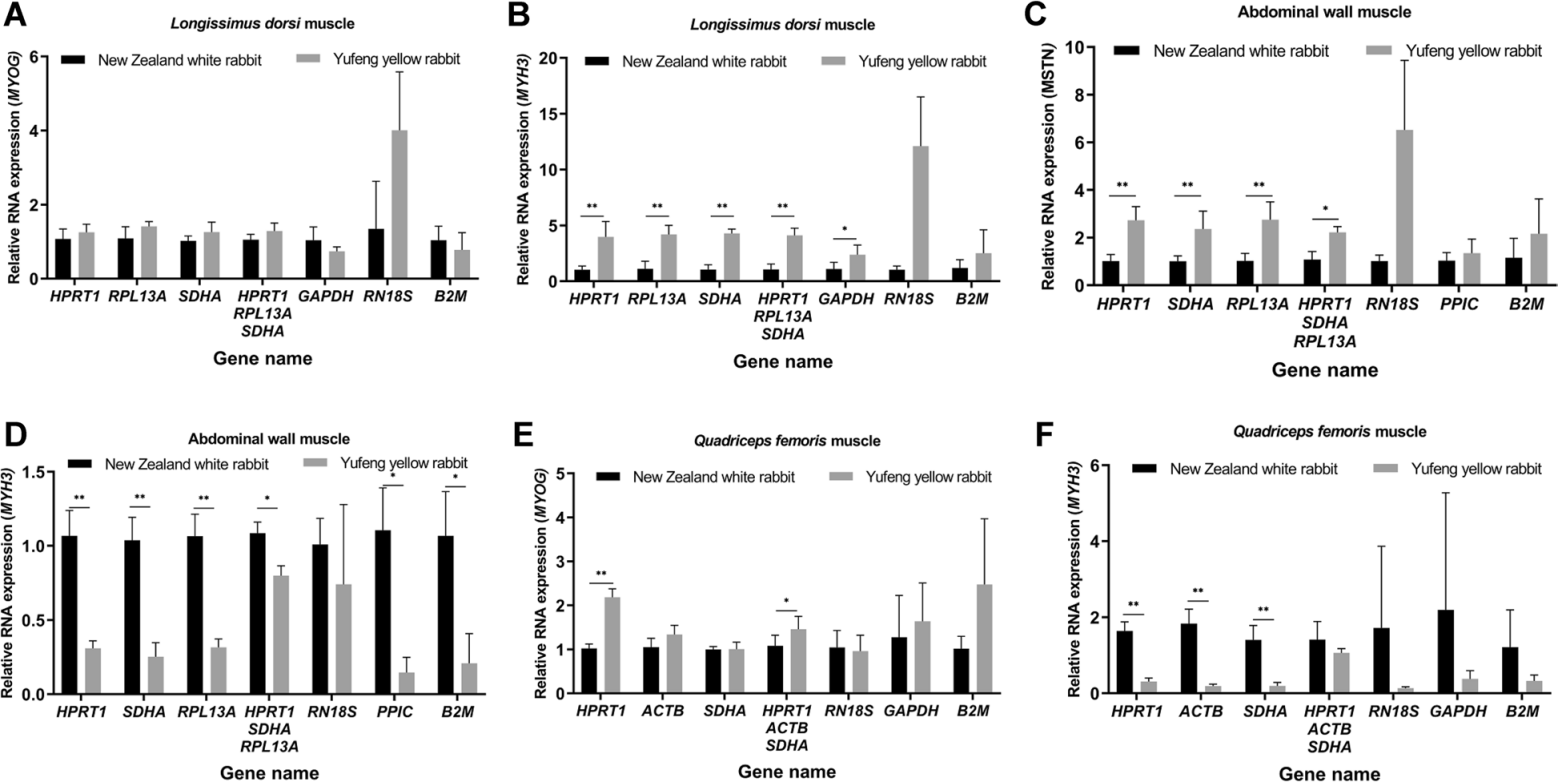
To validate the most suitable reference gene with myogenic regulatory genes. The relative expression of *MYOG* (**A**) and *MYH3* (**B**) in the *longissimus dorsi* muscle. The relative expression of *MSTN* (**C**) and *MYH3* (**D**) in the abdominal wall muscle. The relative expression of *MYOG* (**E**) and *MYH3* (**F**) in the *quadriceps femoris* muscle. * *p* < 0.05, ** *p* < 0.01

To determine whether there is a significant difference in target gene expression normalized to the most stable reference genes versus the least stable reference genes, we analyzed the expression of the top three reference genes normalized to the most stable and their geometric mean as well as the unstable reference genes for *MYOG*, *MYH3* and *MSTN* in different muscle tissues of rabbits. As shown in Fig. 4 and Supplementary Fig. S3, the relative expression patterns of *MYOG*, *MYH3*, and *MSTN* normalized against the *HPRT1*, *ACTB* and *SDHA* genes were basically the same, and the SD value was low. However, the expression patterns were quite different when normalized against the *GAPDH* and *B2M* genes, and the SD value was high. Our results suggested that *HPRT1*, *PPIC* and *SDHA* (the expression of *ACTB* is the most stable only in the *quadriceps femoris* muscle tissue) were the most appropriate reference genes for quantifying target gene expression in rabbit muscle tissues.

## Discussion

In molecular biology studies, gene expression analysis provides a reliable theoretical basis for complex gene regulation research. The 2^-ΔΔCq^ algorithm was utilized to calculate the expression of genes relative to reference genes, and the results calculated with this method depend on the stable expression of reference genes; thus, screening appropriate reference genes is of great importance for accurately normalizing the data and thus obtaining reliable results. However, many studies perform RT-qPCR using reference genes without validation, selecting reference genes by referring to other investigations or arbitrarily selecting commonly used empirical reference genes. The significance of stably expressed reference genes for obtaining accurate RT-qPCR results is always underestimated. Nevertheless, there is almost no reference genes that are stably expressed in different cell types [17, 18] or different tissues of the same species, or under different experimental treatments [19]. The reason for this phenomenon may that different tissues exhibit diverse functions and metabolic characteristics during different developmental periods, and gene expression in tissues varies to different extents to maintain their basal function.

The geNorm, NormFinder and BestKeeper programs are frequently used tools for selecting suitable reference genes, and the stability of reference genes calculated by different programs is different to some extent. According to geNorm software compiled by Vandesompele et al. [12], the appropriate reference genes are screened by Cq value and the optimal number of reference genes are determined. For geNorm software, expression stability is sorted by the expression stability value (M) of candidate reference genes among different samples. The higher the M value, the worse the stability. The expression stability value SV is calculated from the intra-group and inter-group coefficient of variations of each gene between samples according to the NormFinder software compiled by Andersen et al. [13]. The SV value is negatively correlated with the stability of the gene, lower SV value indicates a more stable expression of genes. The standard deviation (SD), coefficient of variation (CV) and correlation coefficient (r) between the Cq values of each candidate reference gene are comprehensively analyzed to screen the best reference gene according to BestKeeper, a software for screening internal reference genes compiled by Pfaffl et al. [14]. Therefore, the results of these programs will be considered comprehensively to select suitable reference genes to prevent possible bias.

This study confirmed that the stable expression of reference genes differed both among different muscle tissues of rabbits and in the same muscle tissue between different breeds of rabbits. However, our comprehensive analysis revealed that the most stable reference genes were *HPRT1*, and it may be selected when a single reference gene is used for RT-qPCR in the muscle tissues of rabbits. When multiple reference genes are selected for calibration, it is recommended that *HPRT1*, *PPIC* and *SDHA* be used together for normalization. *HPRT1* is reported to be involved in cell metabolism; the stability of *HPRT1* has been more commonly recognized, and it has often been selected as a reference gene in recent studies. The *PPIA*, *H2AFZ*, and *HPRT1* genes have been proven to be the most stable reference genes in mouse oocytes and preimplantation-stage embryos under different culture conditions [20], while *HPRT1* and *RPL13A* have been demonstrated to be the most stable reference genes in glioma stem cells (GSCs) [21]. W. Nachar found that both *GAPDH* and *HPRT1* were highly stable reference genes in a rabbit model of left ventricular diastolic dysfunction (LVDD) [22]. By comparing the expression stability of eleven internal reference genes in the skeletal muscle of Meishan pigs and Yorkshire pigs at different ages, Feng found that *HPRT1* presented the highest stability [23].

The expression of *ACTB* gene is stable only in the *quadriceps femoris* muscle. The *ACTB* gene, which encodes actin, is involved in the synthesis of the cytoskeleton structures. The metazoan actin cytoskeleton supports a wide range of contractile and transport processes [24]. Moreover, *ACTB* is a major protein component in striated muscle fibers and a major component in muscle filaments, which plays an important role in cell secretion, migration, cytoplasmic flow and separation, and is highly con-served among different species. Both the *ACTB* and *HPRT1* genes are stably expressed in different tissues of pigs [25], and *ACTB* shows stable expression in the feline endometrium [26]. However, recent studies have shown that *ACTB*, *GAPDH* and *RN18S* are genes with highly variable expression and that their use as reference genes in different tissues of *Capra hircus* should be avoided [27, 28].

The *GAPDH* gene is the most commonly used reference gene for correcting the expression of target genes. However, an increasing number of studies have shown that *GAPDH* is not a stable reference gene [3, 29, 30]. Based on our analysis of the data from three muscle tissues of these two breeds of rabbits, the *ACTB* and *HPRT1* genes were ranked at the top two genes according to comprehensive gene expression stability analyses, while the *GAPDH* gene was proven to be unstable. *GAPDH* encodes an enzyme that catalyzes glycolysis for energy and carbon molecules [31]. The overexpression of *GAPDH* in the T cell lineage promotes angioimmunoblastic T cell lymphoma through an NF-κB-dependent mechanism [32]. The previously used reference genes *GAPDH* and *RN18S* did not show sufficient stability in this study, and we assume that there are great fluctuations in the expression of *GAPDH* and *RN18S* during the course of muscle development. It was speculated that the *GAPDH* gene participates in important oxidation processes in life activities and that its importance varies in different tissues and developmental stages.

To test this hypothesis, the relative gene expression values of the most stable reference genes and the most unstable reference genes were plotted on the basis of normalization against to the geometric average values of *MYOG*, *MYH3*, and *MSTN* in muscles. *MYH3* is a major contractile protein in muscle tissue [33]. *MYOG* is a myogenic regulatory factor that plays a key role in myoblast differentiation [34]. MSTN, a myokine that has attracted recent attention, is a glycoprotein that is secreted primarily by skeletal muscles and acts as a regulator that negatively affects muscle proliferation by suppressing myoblast and skeletal muscle cell proliferation and inhibiting muscle protein synthesis [35, 36]. We observed that the relative expression patterns of these myogenic genes normalized by the unstable reference genes (*GAPDH* and *B2M*) were significantly different from those that normalized by *HPRT1* and *SDHA*. It was apparent that the expression profile showed a similar trend among the stable reference genes, and the results that we obtained were in line with expectations.

## Conclusion

In summary, our data indicated that the optimal reference genes differed among various muscle tissues and between different breeds of rabbits. The results of our comprehensive analysis demonstrated that *HPRT1*, *PPIC* and *SDHA* were the most stable reference genes, and combined analysis of these three reference genes can be used for RT-qPCR normalization in the muscle tissues of rabbits, while the *HPRT1* gene is a suitable reference gene for assessing target gene expression on the basis of only one reference gene. This work assessed the stability of reference genes in muscle tissues of rabbits and will provide a solid foundation for further studies of molecular regulation in the muscle tissues of rabbits.

## Supplementary Information

**Additional file 1: Suppl. Table S1.** Name, accession number, sequences, amplicon length of primer pairs used in the present experiment, and references. **Suppl. Table S2.** Standard curve parameters for the candidate genes. **Fig. S1.** Agarose gel electrophoresis of target products. Marker (left): DL2000 (DGSBio, Guangzhou, China). Marker (right): DL2000 (Vazyme, Nanjing, China). **Fig. S2.** Every gene displayed a single peak profile in the melting curve analysis. -d (RFU)/dT: Negative derivative ratio of fluorescent signal to temperature. **Fig. S3.** To validate the most suitable reference gene with myogenic regulatory genes. The relative expression of *MSTN* (A) in the *longissimus dorsi* muscle. The relative expression of *MYOG* (B) in the abdominal wall muscle. The relative expression of *MSTN* (C) in the *quadriceps femoris* muscle. The relative expression of *MYOG* (D), *MYH3* (E), and *MSTN* (F) in New Zealand white rabbits. The relative expression of *MYOG* (G), *MYH3* (H), and *MSTN* (I) in Yufeng yellow rabbits. * *p* < 0.05, ** *p* < 0.01. **Fig. S4.** Agarose gel electrophoresis of RNA. Marker: DL2000 (DGSBio, Guangzhou, China); 1 and 4: the *longissimus dorsi* muscle tissue; 2 and 5: the abdominal wall muscle tissue; 3 and 6: the *quadriceps femoris* muscle tissue. **Fig. S5.** Optimal number of reference genes for normalization under different experimental conditions. Vn/Vn + 1 indicates the pairwise variation (V) between two sequential normalization factors (NFn and NFn + 1), used to determine the optimal combination of reference genes required for accurate normalization. Values less than 0.15 suggest that another reference gene will not be required for the normalization of gene expression. Pairwise variation analysis (V) to determine the optimal number of reference genes for data normalization in three muscle tissues of New Zealand white rabbits (A), three muscle tissues of Yufeng yellow rabbits (B), the *longissimus dorsi* muscle tissue of New Zealand white rabbits and Yufeng yellow rabbits (C), the abdominal wall muscle tissue of New Zealand white rabbits and Yufeng yellow rabbits (D), and the *quadriceps femoris* muscle tissue of New Zealand white rabbits and Yufeng yellow rabbits (E).

**Additional file 2.**

## Data Availability

The datasets used and analysed during the current study available from the corresponding author on reasonable request.

## Abbreviations

RT-qPCR: Reverse transcription quantitative real-time polymerase chain reaction
MIQE: the Minimum Information for Publication of Quantitative Real-Time PCR Experiments
*ACTB*: Actin beta
*PPIC*: Peptidylprolyl isomerase C
*GAPDH*: Glyceraldehyde-3-phosphate dehydrogenase
*B2M*: Beta-2-microglobulin
*SDHA*: Succinate dehydrogenase complex flavoprotein subunit A
*HPRT1*: Hypoxanthine phosphoribosyltransferase 1
*RPL13A*: Ribosomal protein L13a
*RN18S*: 18S ribosomal RNA
*MYOG*: Myogenin
*MYH3*: Myosin heavy chain 3
*MSTN*: Myostatin
R: Correlation coefficient,
SD: Standard deviation
Cq: Cycle quantification value
