# Supplementary material for: Selection and validation of reference genes for the normalization of quantitative real-time PCR in different muscle tissues of rabbits
Source: BMC Zool. 2022 Dec 15;7:60. doi: 10.1186/s40850-022-00159-0 (PMC10127086; doi:10.1186/s40850-022-00159-0)
Supplement: Supplementary file 1 — Additional file 1: Suppl. Table S1. Name, accession number, sequences, amplicon length of primer pairs used in the present experiment, and references. Suppl. Table S2. Standard curve parameters for the candidate genes. Fig. S1. Agarose gel electrophoresis of target products. Marker (left): DL2000 (DGSBio, Guangzhou, China). Marker (right): DL2000 (Vazyme, Nanjing, China). Fig. S2. Every gene displayed a single peak profile in the melting curve analysis. -d (RFU)/dT: Negative derivative ratio of fluorescent signal to temperature. Fig. S3. To validate the most suitable reference gene with myogenic regulatory genes. The relative expression of MSTN (A) in the longissimus dorsi muscle. The relative expression of MYOG (B) in the abdominal wall muscle. The relative expression of MSTN (C) in the quadriceps femoris muscle. The relative expression of MYOG (D), MYH3 (E), and MSTN (F) in New Zealand white rabbits. The relative expression of MYOG (G), MYH3 (H), and MSTN (I) in Yufeng yellow rabbits. * p < 0.05, ** p < 0.01. Fig. S4. Agarose gel electrophoresis of RNA. Marker: DL2000 (DGSBio, Guangzhou, China); 1 and 4: the longissimus dorsi muscle tissue; 2 and 5: the abdominal wall muscle tissue; 3 and 6: the quadriceps femoris muscle tissue. Fig. S5. Optimal number of reference genes for normalization under different experimental conditions. Vn/Vn + 1 indicates the pairwise variation (V) between two sequential normalization factors (NFn and NFn + 1), used to determine the optimal combination of reference genes required for accurate normalization. Values less than 0.15 suggest that another reference gene will not be required for the normalization of gene expression. Pairwise variation analysis (V) to determine the optimal number of reference genes for data normalization in three muscle tissues of New Zealand white rabbits (A), three muscle tissues of Yufeng yellow rabbits (B), the longissimus dorsi muscle tissue of New Zealand white rabbits and Yufeng yellow rabbits (C), the ab [file 40850_2022_159_MOESM1_ESM.docx]

**Suppl. Table S1** Name, accession number, sequences, amplicon length of primer pairs used in the present experiment, and references.

| **Gene/Acc. #** | **Primers^1^** | **Sequence (5' to 3')** | **bp^2^** | **Reference.** |
| --- | --- | --- | --- | --- |
| *ACTB* | F. 1012 | ATGCAGAAGGAGATCACCGC | 148 | This manuscript |
| *NM_001101683.1* | R. 1159 | ACTCCTGCTTGCTGATCCAC |  |  |
| *HPRT1*  *NM_001105671.1* | F. 237  R. 332 | ACGTCGAGGACTTGGAAAGGGTGTT GGCCTCCCATCTCCTTCATCACATC | 96 | Mamo S et al., 2008 |
| *SDHA* | F. 1676 | CTACGGAGACCTGAAGCACC | 185 | This manuscript |
| *XM_017339216.1* | R. 1860 | ACTCATCGATCCGCACCTTG |  |  |
| *RPL13A XM_002723915.3* | F. 721  R. 802 | CGAGGGCATCAACATTTCCG GTTGGTGTTCATCCGCTTGC | 82 | This manuscript |
| *GAPDH*  *NM_001082253.1* | F. 446  R. 594 | CGATGCCCCCATGTTTGTGA  TCATGAGCCCCTCCACAATG | 149 | This manuscript |
| *RN18S ENSOCUT00000030594.2* | F. 1051  R. 1206 | ATCAGATACCGTCGTAGTTC TTCCGTCAATTCCTTTAAG | 155 | Peng et al., 2012 |
| *PPIC*  *XM_008254951.2* | F. 279  R. 441 | GGGAGAGAGAGGATATGGATAC AATGCCATAGTGCTTCAGC | 163 | Peng et al., 2012 |
| *B2M* | F. 198 | TGTATCTGGGTTTCATCCGCC | 173 | This manuscript |
| *XM_002717921.1* | R. 370 | GTTTCACCCGGCAGGAATAC |  |  |
| *MYOG* | F. 381 | GCCATCCAGTACATCGAGCG | 225 | This manuscript |
| *NM_001177749.1* | R. 605 | CAGGTTGTGGGCGTCTGTAG |  |  |
| *MYH3* | F. 2834 | CTGGAGGATGAGTGTTCGGAG | 127 | This manuscript |
| *NM_001195656.1* | R. 2960 | GGCCAGCAAGCTCCTCAGTAA |  |  |
| *MSTN* | F. 579 | ACTTGACATGAACCCAGGCA | 157 | This manuscript |
| *NM_001109821.1* | R. 735 | TCCTGGTCCTGGGAAGGTTA |  |  |

^1^Primer direction (F-forward, R-reverse) and hybridization position on the sequence.

^2^Amplicon size in base pair (bp)

**Suppl. Table S2** Standard curve parameters for the candidate genes.

| **Genes** | **Slope (m)** | **Intercept (b)** | **Efficiency (E)** | **Correlation (R^2^)** |
| --- | --- | --- | --- | --- |
| *ACTB* | -3.143 | 26.850 | 108.0 | 0.996 |
| *HPRT1* | -3.031 | 32.070 | 113.7 | 0.997 |
| *SDHA* | -3.225 | 27.409 | 104.2 | 0.996 |
| *RPL13A* | -3.228 | 27.758 | 104.1 | 0.994 |
| *GAPDH* | -3.416 | 21.872 | 96.2 | 1.000 |
| *RN18S* | -3.079 | 15.444 | 111.2 | 0.980 |
| *PPIC* | -3.222 | 31.185 | 104.3 | 0.994 |
| *B2M* | -3.095 | 28.995 | 110.4 | 0.997 |
| *MYOG* | -3.361 | 24.070 | 98.3 | 0.995 |
| *MYH3* | -3.392 | 28.196 | 97.2 | 0.980 |
| *MSTN* | -3.231 | 24.292 | 103.9 | 0.996 |


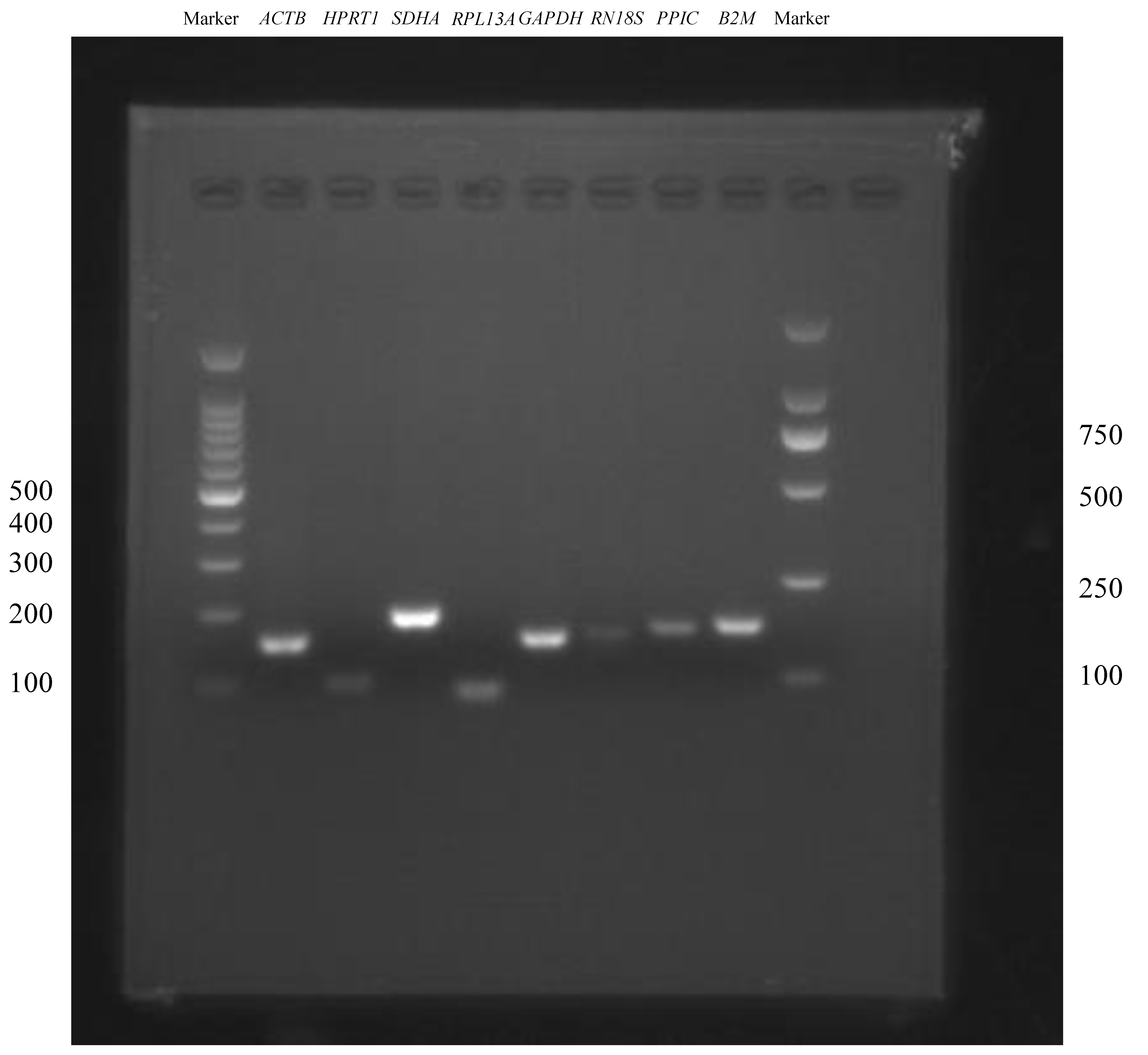


**Fig. S1** Agarose gel electrophoresis of target products. Marker (left): DL2000 (DGSBio, Guangzhou, China). Marker (right): DL2000 (Vazyme, Nanjing, China).


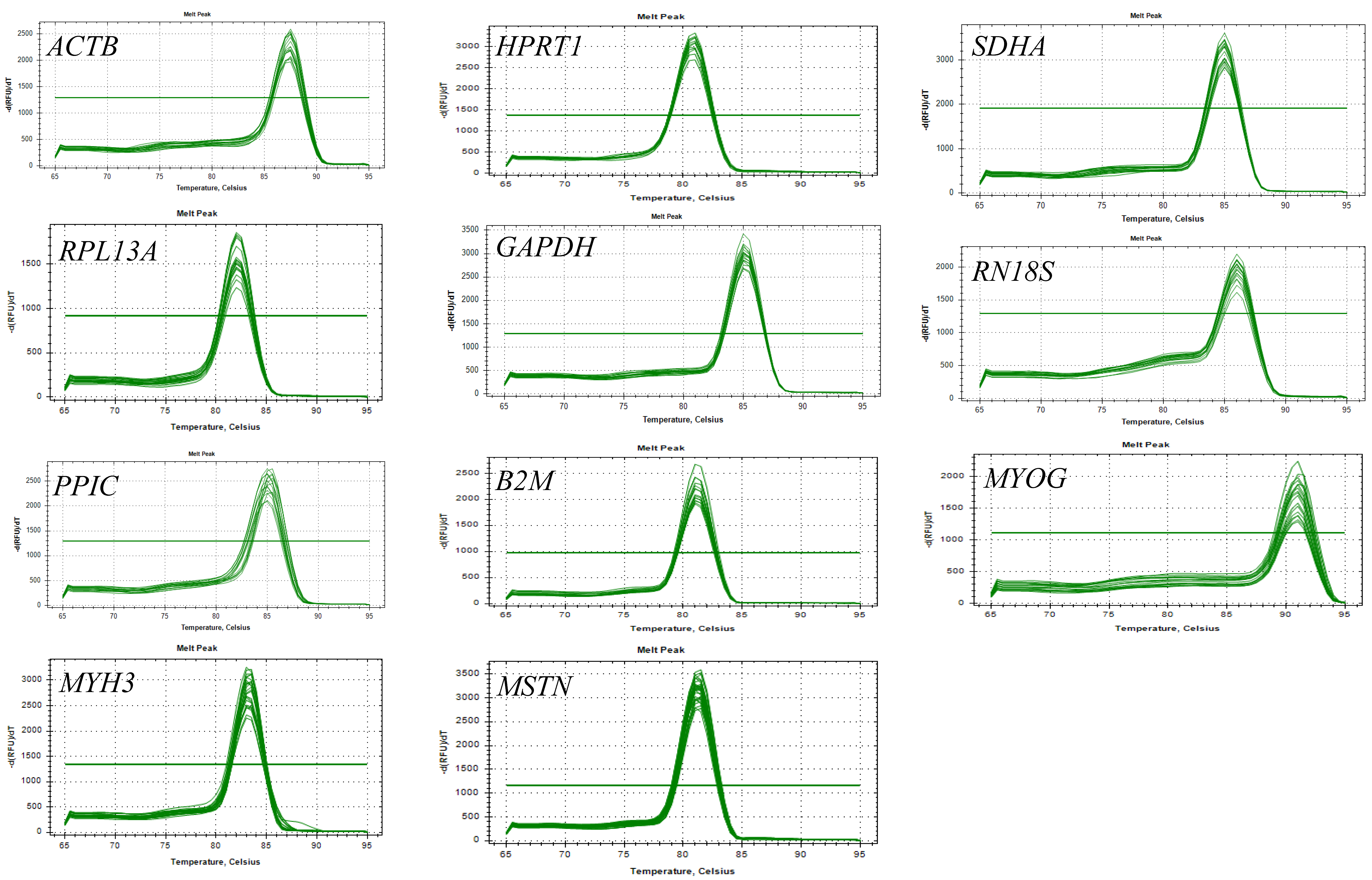


**Fig. S2** Every gene displayed a single peak profile in the melting curve analysis. -d(RFU)/dT: Negative derivative ratio of fluorescent signal to temperature.


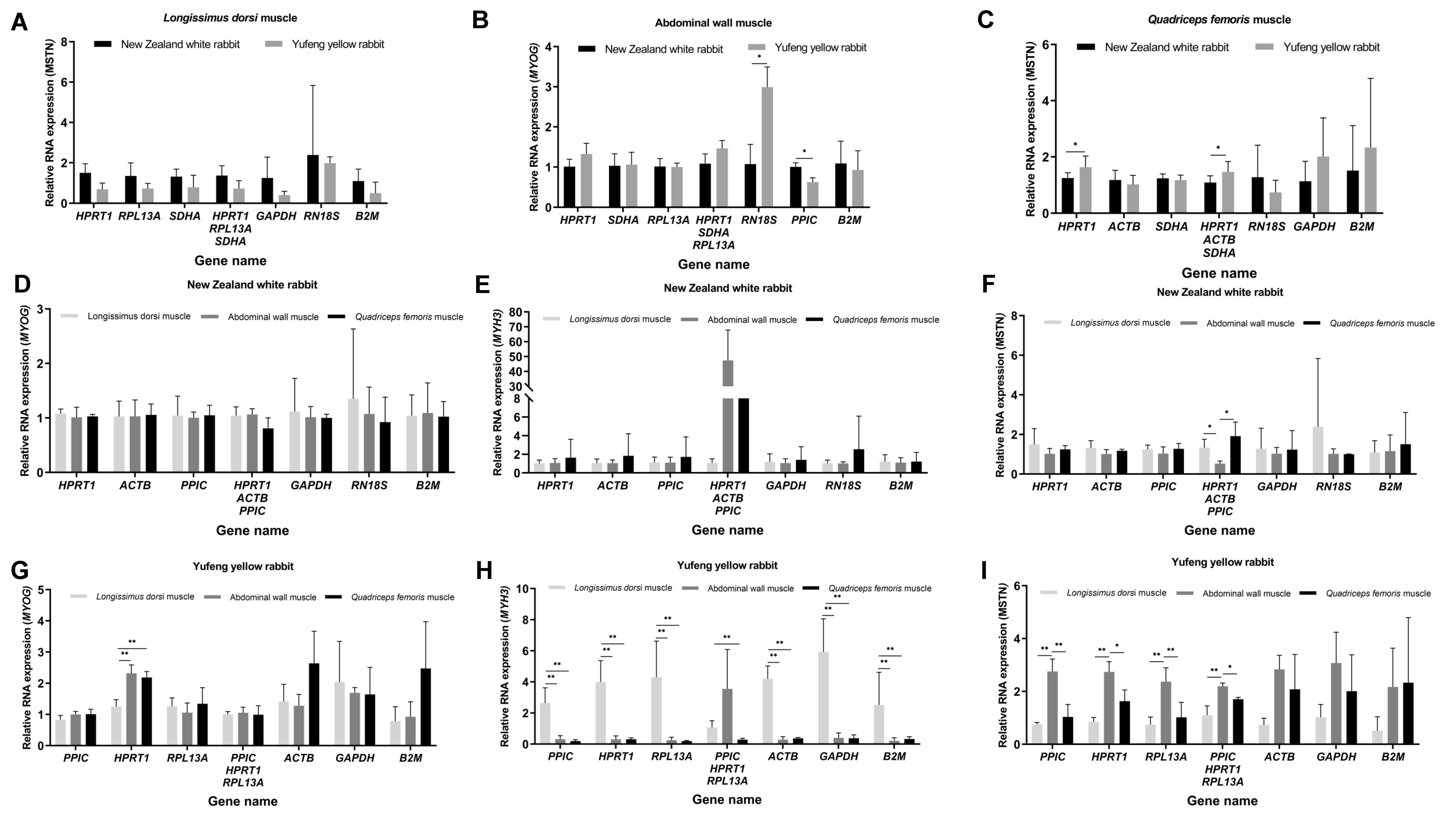


**Fig. S3** To validate the most suitable reference gene with myogenic regulatory genes. The relative expression of *MSTN* **(A)** in the *longissimus dorsi* muscle. The relative expression of *MYOG* **(B)** in the abdominal wall muscle. The relative expression of *MSTN* **(C)** in the *quadriceps femoris* muscle. The relative expression of *MYOG* **(D)**, *MYH3* **(E)**, and *MSTN* **(F)** in New Zealand white rabbits. The relative expression of *MYOG* **(G)**, *MYH3* **(H)**, and *MSTN* **(I)** in Yufeng yellow rabbits. * *p* < 0.05, ** *p* < 0.01.


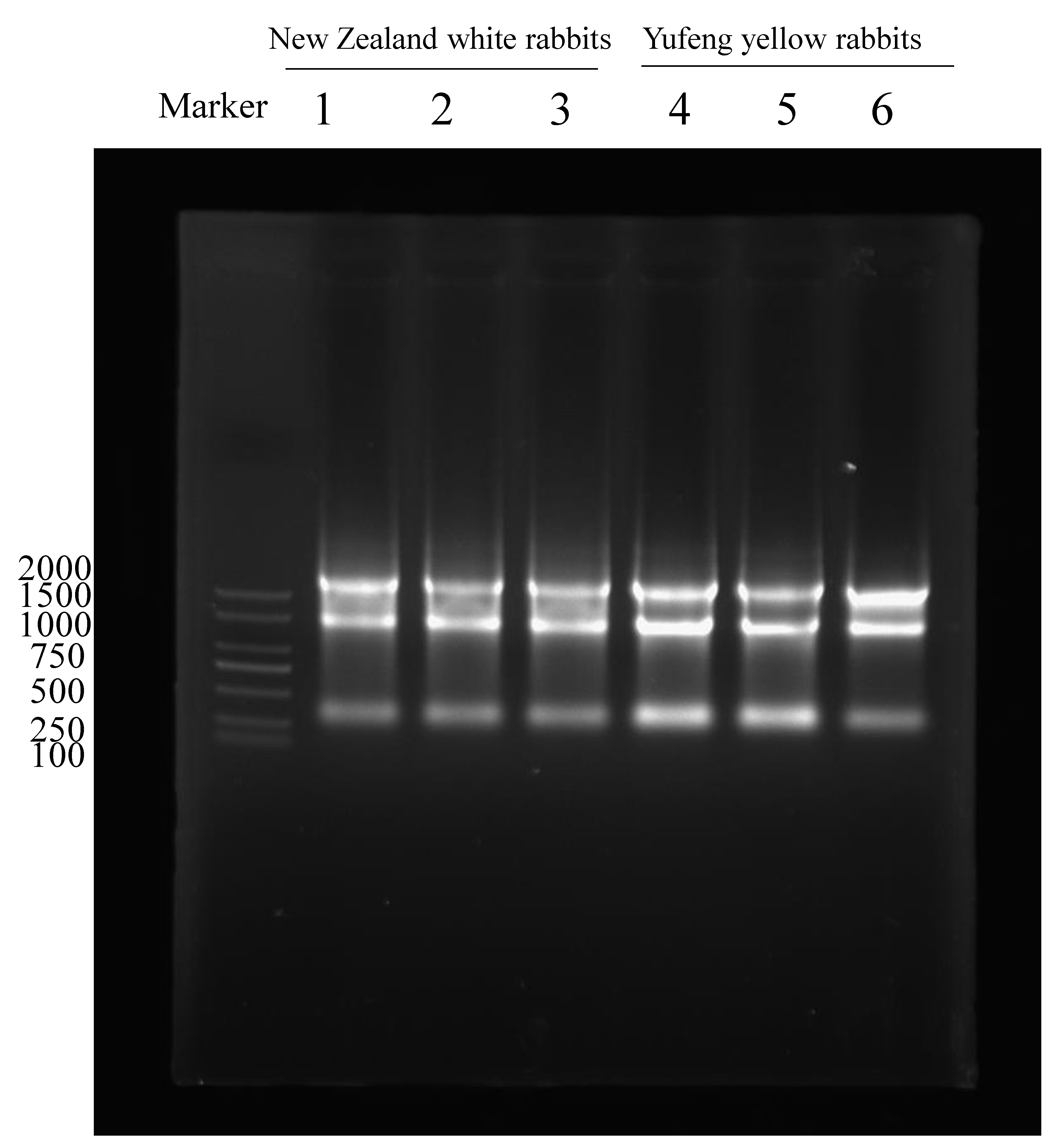


**Fig. S4** Agarose gel electrophoresis of RNA. Marker: DL2000 (DGSBio, Guangzhou, China); 1 and 4: the *longissimus dorsi* muscle tissue; 2 and 5: the abdominal wall muscle tissue; 3 and 6: the *quadriceps femoris* muscle tissue.


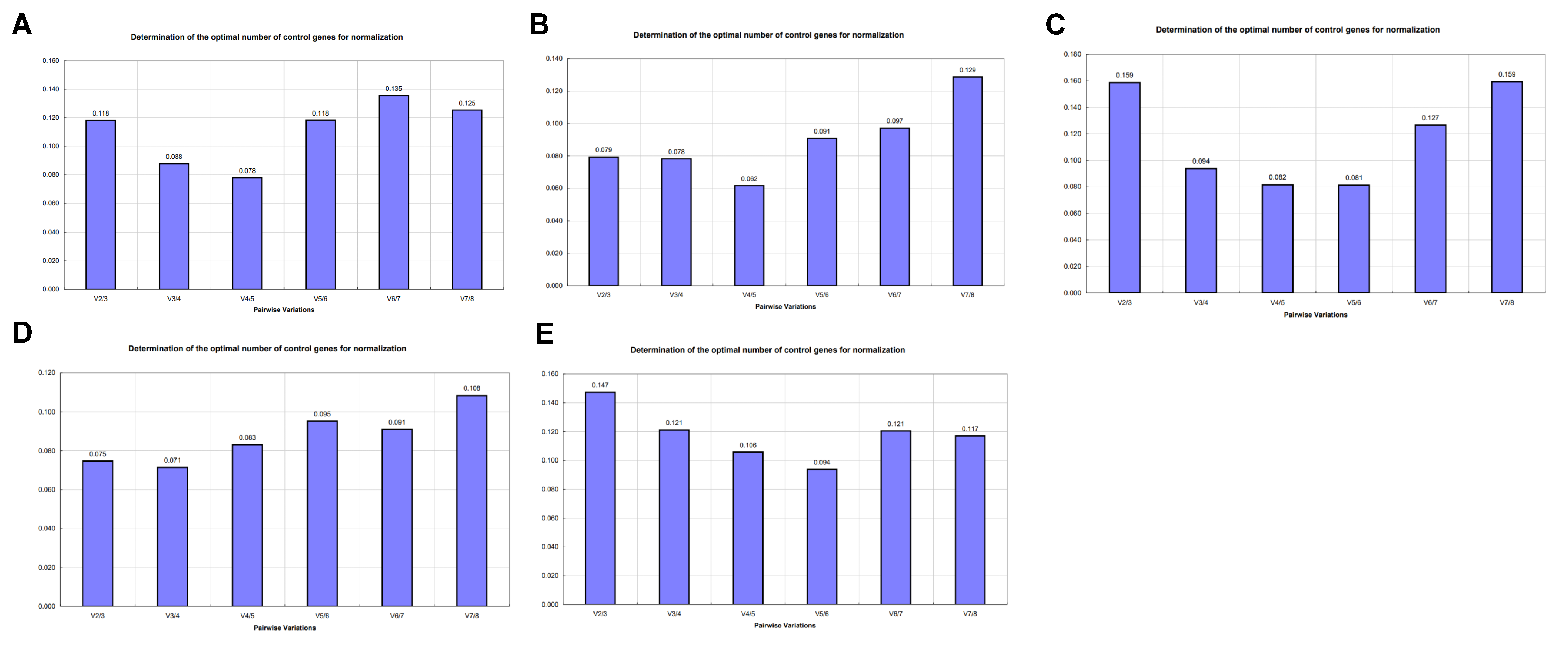


**Fig. S5** Optimal number of reference genes for normalization under different experimental conditions. Vn/Vn+1 indicates the pairwise variation (V) between two sequential normalization factors (NFn and NFn+1), used to determine the optimal combination of reference genes required for accurate normalization. Values less than 0.15 suggest that another reference gene will not be required for the normalization of gene expression. Pairwise variation analysis (V) to determine the optimal number of reference genes for data normalization in three muscle tissues of New Zealand white rabbits (A), three muscle tissues of Yufeng yellow rabbits (B), the *longissimus dorsi* muscle tissue of New Zealand white rabbits and Yufeng yellow rabbits (C), the abdominal wall muscle tissue of New Zealand white rabbits and Yufeng yellow rabbits (D), and the *quadriceps femoris* muscle tissue of New Zealand white rabbits and Yufeng yellow rabbits (E).
