# Supplementary material for: Selection and validation of reference genes for the normalization of quantitative real-time PCR in different muscle tissues of rabbits
Source: BMC Zool. 2022 Dec 15;7:60. doi: 10.1186/s40850-022-00159-0 (PMC10127086; doi:10.1186/s40850-022-00159-0)
Supplement: Supplementary file 2 — Additional file 2. [file 40850_2022_159_MOESM2_ESM.docx]

| Evaluation and validation of selected reference genes | | | |
| --- | --- | --- | --- |
| Items | Gene  Name | Levene | T/ANOVA-TEST |
|  |  | F | Significance |
| The longissimus dorsi muscle (MYOG) | HPRT1 | 0.112456 | 0.746 |
|  | RPL13A | 0.707629 | 0.424661 |
|  | ACTB | 2.161575 | 0.18 |
|  | HPRT1& RPL13A& ACTB | 0.6397576 | 0.446896 |
|  | CYP | 1.7872761 | 0.218026 |
|  | RN18S | 0.0138953 | 0.90907 |
|  | B2M | 0.0720758 | 0.795131 |
| The longissimus dorsi muscle (MYH3) | HPRT1 | 2.8056554 | 0.132463 |
|  | RPL13A | 0.0874703 | 0.774949 |
|  | ACTB | 0.0017234 | 0.967904 |
|  | HPRT1& RPL13A& ACTB | 0.1504987 | 0.708178 |
|  | CYP | 0.4862771 | 0.50535 |
|  | RN18S | 3.9294344 | 0.082756 |
|  | B2M | 1.9782028 | 0.197216 |
| The longissimus dorsi muscle (MSTN) | HPRT1 | 0.4381129 | 0.526637 |
|  | RPL13A | 1.6670315 | 0.232713 |
|  | ACTB | 0.686482 | 0.431387 |
|  | HPRT1& RPL13A& ACTB | 0.110464 | 0.748158 |
|  | CYP | 3.6449053 | 0.092651 |
|  | RN18S | 4.0801926 | 0.078065 |
|  | B2M | 0.0314916 | 0.863559 |
| The abdominal wall muscle (MYOG) | HPRT1 | 0.2453083 | 0.633714 |
|  | ACTB | 0.0611591 | 0.810902 |
|  | SDHA | 2.2558647 | 0.171509 |
|  | HPRT1& ACTB& SDHA | 0.2318756 | 0.643033 |
|  | RN18S | 0.0170541 | 0.899323 |
|  | CYP | 2.233E-07 | 0.999635 |
|  | B2M | 0.0268523 | 0.8739 |
| The abdominal wall muscle (MYH3) | HPRT1 | 3.7081924 | 0.090323 |
|  | ACTB | 1.4589672 | 0.261587 |
|  | SDHA | 1.3927475 | 0.271841 |
|  | HPRT1& ACTB& SDHA | 0.0140933 | 0.908428 |
|  | RN18S | 2.8896599 | 0.127572 |
|  | CYP | 2.42751 | 0.157838 |
|  | B2M | 0.6986393 | 0.427499 |
| The abdominal wall muscle (MSTN) | HPRT1 | 1.7420201 | 0.223398 |
|  | ACTB | 2.6209521 | 0.144121 |
|  | SDHA | 1.785487 | 0.218235 |
|  | HPRT1& ACTB& SDHA | 0.5396313 | 0.483559 |
|  | RN18S | 4.0253595 | 0.07973 |
|  | CYP | 0.8827517 | 0.374947 |
|  | B2M | 0.7404878 | 0.414548 |
| The quadriceps femoris muscle (MYOG) | HPRT1 | 0.7241302 | 0.419532 |
|  | ACTB | 0.00819 | 0.930116 |
|  | SDHA | 2.7755741 | 0.134274 |
|  | HPRT1& ACTB& SDHA | 0.1288423 | 0.728924 |
|  | CYP | 0.0100128 | 0.922756 |
|  | GAPDH | 0.0122065 | 0.914748 |
|  | B2M | 3.0999986 | 0.116326 |
| The quadriceps femoris muscle (MYH3) | HPRT1 | 3.1477369 | 0.113957 |
|  | ACTB | 1.0757825 | 0.329974 |
|  | SDHA | 2.1945452 | 0.176777 |
|  | HPRT1& ACTB& SDHA | 2.5572286 | 0.148458 |
|  | CYP | 4.9526853 | 0.056697 |
|  | GAPDH | 4.3344577 | 0.070901 |
|  | B2M | 4.277583 | 0.072428 |
| The quadriceps femoris muscle (MSTN) | HPRT1 | 1.5398984 | 0.249787 |
|  | ACTB | 0.077451 | 0.78784 |
|  | SDHA | 0.20428 | 0.663291 |
|  | HPRT1& ACTB& SDHA | 0.8433997 | 0.385278 |
|  | CYP | 2.0584459 | 0.189278 |
|  | GAPDH | 0.8261798 | 0.389941 |
|  | B2M | 0.3977468 | 0.545846 |
| New Zealand white rabbits (MYOG) | HPRT1 | 3.3328109 | 0.10631 |
|  | ACTB | 0.2802152 | 0.764992 |
|  | CYP | 1.4755072 | 0.301188 |
|  | HPRT1& ACTB& CYP | 0.8261155 | 0.482047 |
|  | SDHA | 2.8415692 | 0.135449 |
|  | RN18S | 3.5140621 | 0.097681 |
|  | B2M | 0.8361489 | 0.478275 |
| New Zealand white rabbits (MYH3) | HPRT1 | 3.6933724 | 0.090039 |
|  | ACTB | 3.4808132 | 0.099192 |
|  | CYP | 2.2632161 | 0.185187 |
|  | HPRT1& ACTB& CYP | 3.7710128 | 0.086977 |
|  | SDHA | 2.7770099 | 0.140041 |
|  | RN18S | 10.489648 | 0.010999 |
|  | B2M | 0.9456919 | 0.439536 |
| New Zealand white rabbits (MSTN) | HPRT1 | 4.9149342 | 0.054453 |
|  | ACTB | 3.7950954 | 0.086055 |
|  | CYP | 0.2776321 | 0.766802 |
|  | HPRT1& ACTB& CYP | 4.2069901 | 0.072128 |
|  | SDHA | 1.9410545 | 0.223823 |
|  | RN18S | 11.654708 | 0.008579 |
|  | B2M | 2.7078075 | 0.145196 |
| Yufeng yellow rabbits (MYOG) | SDHA | 0.6097721 | 0.574017 |
|  | HPRT1 | 4.4354946 | 0.06568 |
|  | ACTB | 1.4150099 | 0.313739 |
|  | SDHA& HPRT1& ACTB | 2.0747563 | 0.206594 |
|  | RPL13A | 2.9857773 | 0.125893 |
|  | GAPDH | 3.4761217 | 0.099407 |
|  | B2M | 3.740538 | 0.088162 |
| Yufeng yellow rabbits (MYH3) | SDHA | 1.4399159 | 0.308489 |
|  | HPRT1 | 2.3110312 | 0.18023 |
|  | ACTB | 5.7150116 | 0.040791 |
|  | SDHA& HPRT1& ACTB | 3.7847498 | 0.086449 |
|  | RPL13A | 3.0219661 | 0.123637 |
|  | GAPDH | 9.0194123 | 0.015549 |
|  | B2M | 4.0142445 | 0.078239 |
| Yufeng yellow rabbits (MSTN) | SDHA | 4.2952522 | 0.069541 |
|  | HPRT1 | 1.144938 | 0.379149 |
|  | ACTB | 1.021649 | 0.415099 |
|  | SDHA& HPRT1& ACTB | 1.9068465 | 0.228537 |
|  | RPL13A | 1.1089588 | 0.389196 |
|  | GAPDH | 1.2822118 | 0.343842 |
|  | B2M | 3.4588973 | 0.100205 |
